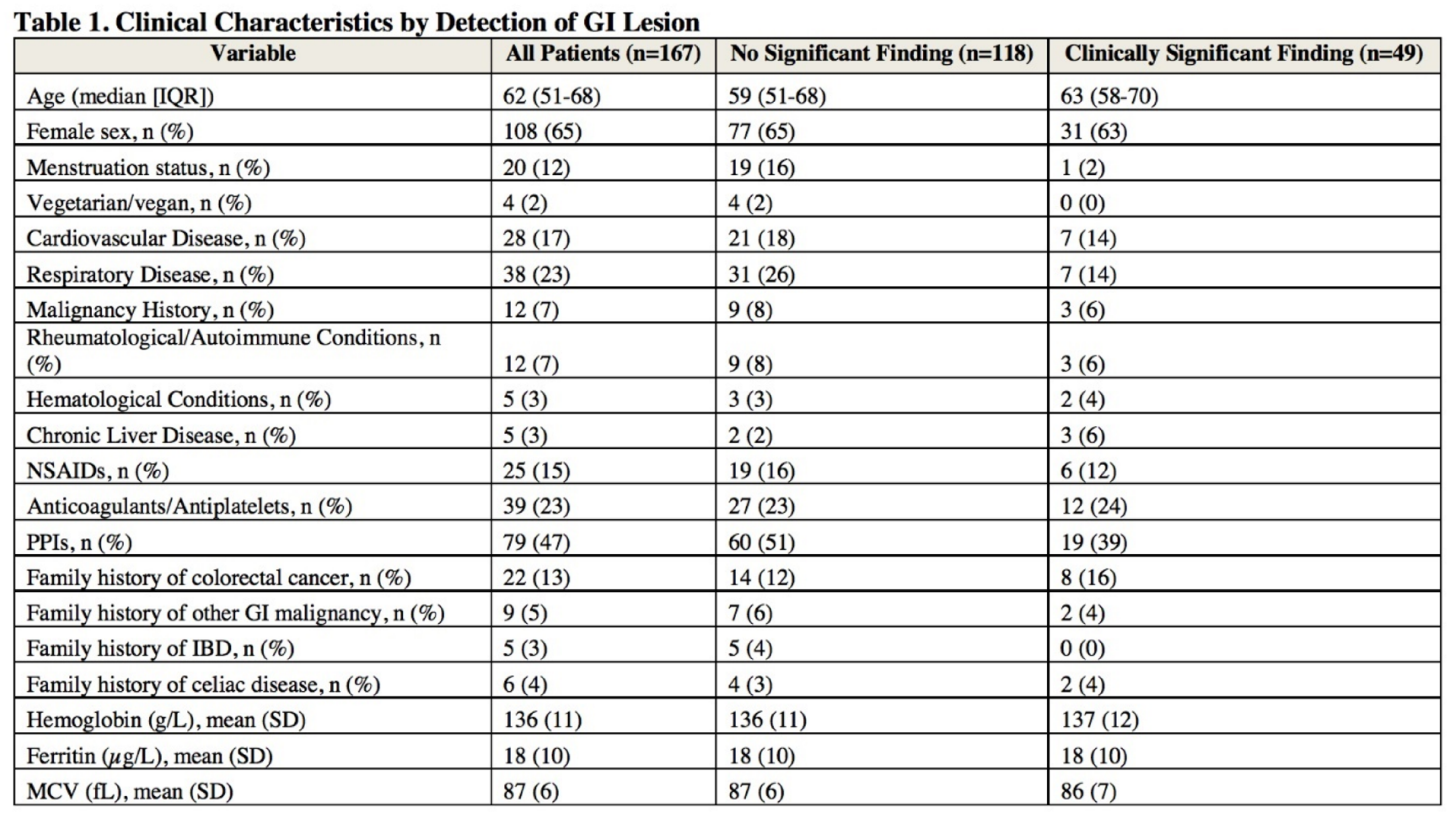# Poster Session I - A147 ENDOSCOPIC FINDINGS IN NON-ANEMIC IRON DEFICIENCY

**DOI:** 10.1093/jcag/gwaf042.147

**Published:** 2026-02-13

**Authors:** L Huynh, E Squirell

**Affiliations:** Queen’s University, Kingston, ON, Canada; Queen’s University, Kingston, ON, Canada

## Abstract

**Background:**

Bidirectional endoscopy is recommended by major gastroenterology associations as part of the initial assessment of iron-deficiency anemia to rule out gastrointestinal (GI) pathology such as malignancy. However, current guidelines are less clear on the role of endoscopic evaluation in non-anemic iron deficiency (NAID). To date, there are no North American data describing the rate of clinically significant GI lesions in this population.

**Aims:**

We aimed to determine the rates and etiologies of clinically significant GI lesions detected on bidirectional endoscopy in NAID, and to identify clinical risk factors associated with lesion detection.

**Methods:**

We conducted a single-centre cross-sectional study of adults aged 18 or older who underwent elective outpatient bidirectional endoscopy for NAID at Kingston Health Sciences Centre between November 2019 and November 2024. Data were collected by retrospective chart review, including patient demographics, comorbidities, family history, medication history, iron indices, and menstrual status. The primary outcome was the detection of a GI lesion known to contribute to iron deficiency on bidirectional endoscopy. Multivariate logistic regression was used to assess factors associated with lesion detection after controlling for age, sex, menstruation status, mean corpuscular volume (MCV), and PPI use.

**Results:**

A total of 167 patients were included in our study (median age 62 years [IQR 51-68]), range 18-91; 65% female; 19% with family history of GI malignancy). Clinically significant findings were identified in 49 patients (29%), including: malignancy (n = 4, 2%), inflammatory bowel disease (IBD) (n = 4, 2%), celiac disease (n = 7, 4%), vascular lesion (n = 2, 1%), peptic ulcer disease (n = 4, 2%), gastritis (NSAID, H. pylori, or autoimmune related) (n-=15, 9%), Cameron’s erosions (n = 3, 2%), advanced polyp (n = 3, 2%), marginal ulcer (n = 1, 1%), portal hypertensive changes (n = 3, 2%), and severe erosive esophagitis (n = 4, 2%). 1 of the 49 patients had both IBD and Cameron’s erosions detected. On multivariate analysis, menstruation status (OR 0.10, 95% CI 0.01-0.98) and PPI use (OR 0.46, 95% CI 0.22-0.95) were associated with lower odds of detecting endoscopic findings.

**Conclusions:**

Clinically significant endoscopic findings are common among patients investigated for NAID and mostly driven by gastritis, however the rate of detecting malignant lesions is low. Pre-menopausal patients with NAID were less likely to have findings detected on endoscopy, suggesting that endoscopic investigations could be avoided in this patient population in the absence of other risk factors. Patients with NAID should be evaluated for further endoscopic investigation after an informed discussion of the risks and benefits and consideration of the patient’s age and medical comorbidities.

**Funding Agencies:**

None